# Subcutaneous immunoglobulins replacement therapy in secondary antibody deficiencies: Real life evidence as compared to primary antibody deficiencies

**DOI:** 10.1371/journal.pone.0247717

**Published:** 2021-03-04

**Authors:** Francesco Cinetto, Raffaella Neri, Fabrizio Vianello, Andrea Visentin, Gregorio Barilà, Sabrina Gianese, Alison Lanciarotta, Cinzia Milito, Marcello Rattazzi, Francesco Piazza, Livio Trentin, Renato Zambello, Carlo Agostini, Riccardo Scarpa

**Affiliations:** 1 Department of Medicine–DIMED, University of Padua, Padua, Italy; 2 Formerly Haematology and Clinical Immunology Unit, University of Padua, Padua, Italy; 3 Regional Center for Rare Immunological Diseases, Internal Medicine 1, Treviso Hospital, University of Padua, Padua, Italy; 4 Haematology and Clinical Immunology Unit, University of Padua, Padua, Italy; 5 Department of Molecular Medicine, Sapienza University of Rome, Rome, Italy; Mazzini Hospital, ITALY

## Abstract

Secondary antibody deficiencies (SAD) may require immunoglobulin replacement therapy (IgRT). While the intravenous route (IVIG) is broadly considered effective in SAD, the use of subcutaneous immunoglobulins (SCIG) is mainly adopted from the experience in primary antibody deficiencies (PAD), where SCIG have been shown to perform as effective as IVIG. However, evidence-based data on SCIG administration in SAD patients are still insufficient. Herein we retrospectively evaluated the efficacy and safety profile of SCIG treatment in 131 SAD patients as compared to a group of 102 PAD patients. We found SCIG being equally effective in reducing annual infectious rate both in SAD and PAD patients. However, SAD patients required lower SCIG dosage and lower IgG through level to achieve similar biological effect in terms of infection burden, at the steady state. SAD patients also showed better correlation between SCIG dose and serum IgG achieved value. Furthermore, within SAD, SCIG were found to work irrespective of the underlying disease. Especially in Non-Hodgkin Lymphoma patients, whose indication to IgRT is still not included in all guidelines and for whom evidence-based data are still lacking, SCIG were as effective as in Chronic Lymphocytic Leukemia or Multiple Myeloma patients, and SCIG discontinuation, without evidence of B cell recovery, led to IgG decline and relapsed infections. Finally, treatment tolerance in SAD patients was comparable to the PAD cohort. Globally, our data suggest that SCIG, as already appreciated in PAD, represent a valuable option in SAD patients, independent on the disease leading to antibody deficiency.

## Introduction

Humoral immunodeficiency syndromes may occur as primary or secondary abnormalities, with defects in all or in only some classes of immunoglobulins.

The primary antibody deficiency syndromes (PAD) are a group of rare monogenic or polygenic disorders affecting B cell development. Secondary humoral immunodeficiencies are far more prevalent than primary immunodeficiencies and include a wide-range of neoplastic conditions like Chronic Lymphocytic Leukaemia (CLL), Multiple Myeloma (MM), Non-Hodgkin Lymphoma (NHL) [1–4] and thymoma [5] as well as non-neoplastic conditions as solid organ transplantation [6], chronic infectious diseases (especially viral infections) [7], protein loss or drug induced syndromes [8, 9].

Regardless of the pathogenesis, inability to produce functional immunoglobulins both in primary and secondary antibody deficiencies (SAD) is responsible for an increased susceptibility to infections. Therefore, immunoglobulin replacement therapy (IgRT) administered intravenously (IVIG) or subcutaneously (SCIG), represents an invaluable opportunity for minimizing infections and improving health-related quality of life (HRQL) in patients with PAD and SAD.

There is substantial evidence that IVIG are effective in reducing the number of infections, the use of antibiotics as well as hospitalization and loss of working days in SAD [10, 11]. Guidelines from the European Medicines Agency (EMA) have been provided on the use of IVIG in secondary hypogammaglobulinemia in the context of hematologic diseases in patients affected by recurrent infections [12].

On the contrary, the use of SCIG in SAD, is mainly borrowed from their proven efficacy in primary immunodeficiency syndromes, as stated by EMA and other international societies [13–16]. In fact, both SCIG and IVIG replacement therapy represent the standard of care for PAD and this is based on robust and consistent data on the effectiveness and safety of these treatments in this setting [17]. Studies in PAD patients also report improved HRQL following the switch from IVIG to SCIG [18]. A recent meta-analysis of 24 studies involving 946 PAD patients shows that shifting from IVIG to SCIG therapy leads to higher level of IgG and less side effects with no differences in terms of patient infection rate [19]. Of importance, home-based SCIG therapy has been shown also to lead to economic benefits if compared to hospital-based IVIG treatment [20].

Furthermore, there is lack of consistency between different country guidelines with respect to SAD conditions subject to SCIG indication, and also divergence between recommendations and clinical practice, with NHL representing a frequent condition motivating IgRT besides well-recognised indications [21, 22]. Currently, data on direct comparison of IVIG and SCIG in the setting of SAD patients as well as how SCIG perform in SAD compared to PAD are largely insufficient: a recent literature review performed on the worldwide recognised medical databases identified only 7 articles that included SAD patients treated with SCIG replacement therapy [23]. In this paper we aim to retrospectively evaluate and compare the efficacy and safety of SCIG replacement therapy (SCIG RT) in a cohort of patients with PAD and SAD.

## Methods

In this retrospective real-life single-centre study we analysed a cohort of subjects with confirmed diagnosis of PAD following ESID Registry criteria (https://esid.org/Working-Parties/Registry-Working-Party/Diagnosis-criteria), and a cohort of subjects with SAD, referred to or in follow up at our Haematology and Clinical Immunology Unit and treated with SCIG RT.

Inclusion criteria were as follow:

- at least 12 weeks of SCIG RT (we adopted this specific time frame considering the minimum amount of time to achieve target IgG level of 600 mg/dl in the absence of a loading phase [24, 25])
- follow-up of at least 12 months before and after SCIG
- available data on comorbidities, episodes of infection, need for topic, oral or iv antibiotic therapy as well as hospitalisation events, pre- and post-SCIG RT immunoglobulin profiles (IgG, IgA and IgM), treatment-related local site reactions and systemic adverse events

Databases and clinical records consultation were done between February and December 2019. We analysed data about SCIG formulation, dosage and infusion schedule in PAD and SAD cohorts.

Efficacy of SCIG RT in PAD and SAD cohorts was determined based on IgG trough level at the steady state, after at least 12 weeks of therapy, and on the annualized rate of non-neutropenic infection per patient.

Infectious events were defined as serious bacterial infections (SBI) in case of sepsis, pneumonia, osteomyelitis, septic arthritis, visceral abscess, meningitis or endocarditis diagnosed by a practising physician according to standard medical procedures (including physical examination, laboratory tests, bacterial cultures, imaging when appropriated).

Treatment safety was determined based on the rate of local site reactions and systemic adverse events reported in patient medical records.

Continuous variables were assessed as mean, standard deviation, median and interquartile range (IQR). Statistical comparisons by groups were evaluated by specific t-test, depending on the normality of the underlying distribution. In case of three or more groups analysis of variance (ANOVA) test were performed. Linear regression analysis and Pearson correlation coefficient were used to investigate the strength of relationship between dependent and independent variables (software Prism, release 8.3.1, © 2019 GraphPad Software).

The study was approved by the ethics committee for clinical trials of the Provinces of Treviso and Belluno and was conformed to the Helsinki declaration of 1975, as revised in 2013. All patients signed informed consent.

## Results

### Patients

**Fig 1:**
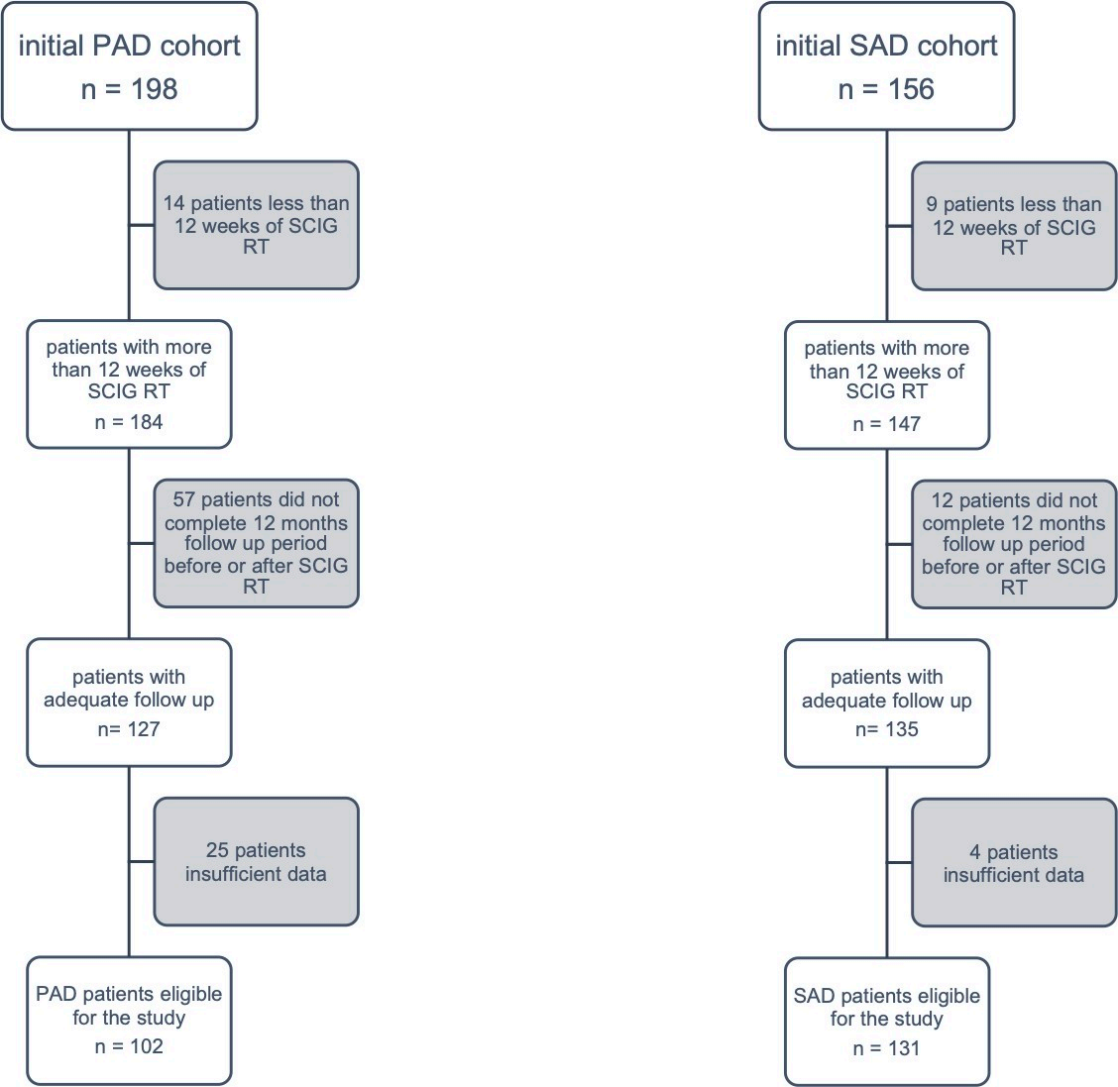
Inclusion criteria in PAD and SAD cohorts.

By analysing the clinical records of the period between September 2011 and February 2018, 198 subjects with PAD and 156 patients with SAD were considered for the study. After excluding patients not fulfilling inclusion criteria (Fig 1), a cohort of 102 subjects with PAD and a cohort of 131 subjects with SAD were considered eligible for the study (Table 1).

**Table 1:** Demographics and clinical history of PAD and SAD patients.

Patients’ characteristics	PAD n = 102	SAD n = 131
Male gender, n (%)	53 (52)	66 (50,4)
Female gender, n (%)	49 (48)	65 (49,6)
Age (years), Mean ± SD	51,6 ± 14,8	70,0 ± 12,7
PAD diagnosis		
CVID, n (%)	75 (73,5)	
Isolated IgG subclass deficiency, n (%)	5 (4,9)	
IgA with IgG subclass deficiency, n (%)	2 (2)	
SPAD, n (%)	1 (1)	
Unclassified antibody deficiency, n (%)	17 (16,6)	
Agammaglobulinemia, n (%)	2 (2)	
SAD diagnosis		
MM, n (%)		22 (16,8)
CLL, n (%)		55 (42)
NHL, n (%)		34 (26)
Others, n (%)		20 (15,2)
SAD disease-specific treatment		
Hematologic malignancy, 110/111 patients		
anti-CD20 monoclonal antibody, n (%)		12 (10,9)
chemotherapy, n (%)		35 (31,8)
anti-CD20 monoclonal antibody + chemotherapy, n (%)		63 (57,3)
Others, 20/20 patients		
anti-CD20 monoclonal antibody, n (%)		5 (25)
anti-CD20 monoclonal antibody + immunosuppressants, n (%)		1 (5)
immunosuppressants, n (%)		14 (70)
SCIG replacement therapy		
Naive, n (%)	53 (52)	99 (75,6)
Previous IVIG, n (%)	49 (48)	32 (24,4)
Duration (months), Mean ± SD	73,8 ± 42,4	45,8 ± 23,3
SCIG formulation		
20%, n (%)	45 (44,1)	75 (57,2)
16 or 16,5%, n (%)	43 (42,2)	54 (41,2)
10% facilitated, n (%)	14 (13,7)	2 (1,6)

Among PAD patients, Common Variable Immunodeficiency (CVID) was the diagnosis in 75/102 patients (of which 54/75 and 21/75 showed combined IgG, IgA and IgM or IgG and IgA reduction, respectively), 5/102 had isolated IgG subclass deficiency, 2/102 had IgA with IgG subclass deficiency, 1 patient had specific deficit of anti-pneumococcal Ig (SPAD), 17/102 had unclassified antibody deficiency (UAD), whereas 2/102 had agammaglobulinemia. Mean age was 51.58 ± 14.81 years. 53/102 patients were females.

SAD group included 111/131 patients affected by hematologic malignancies (55 patients with CLL, 22 with MM and 34 with NHL) and 20/131 patients presenting with hypogammaglobulinemia related to drugs administered for immune-mediated disorders. Mean age was 70.03 ± 12.71 years. 65/131 patients were females and 66/131 were males.

One-hundred and ten out of 111 hematologic patients received a disease-specific treatment before and/or during immunoglobulin replacement therapy. Thirty-five out of 111 received chemotherapy only, 12 out of 111 were treated with anti-CD20 monoclonal antibody alone and 63 out of 111 received anti-CD20 monoclonal antibody in combination with chemotherapy. In addition, 7/111 SAD patients underwent autologous hematopoietic stem cell transplantation (HSCT).

Among the 20 patients with hypogammaglobulinemia secondary to treatment of immune-mediated disorders, 14/20 were treated with immunosuppressants, 5/20 with anti-CD20 monoclonal antibody alone and 1/20 with a combination of both. Demographics and clinical history of PAD and SAD patients are recapitulated in Table 1. Furthermore, additional description of SAD cohort comorbidities and immunoglobulin profiles is reported in S1 Table.

### Immunoglobulin replacement therapy

According to clinical records, IgRT was initiated in SAD subjects with hypogammaglobulinemia (IgG <600 mg/dL) complaining of serious non-neutropenic infectious event, or when an increased incidence of non-neutropenic infections requiring antibiotic therapy was detected (more than 2 episodes in the last 12 months). In PAD patients IgRT was initiated according to current guidelines [17]. SCIG RT were initially administered at similar mean dosage in both PAD and SAD (280.14 ± 84.38 mg/kg/month in PAD and 267.74 ± 59.78 mg/kg/month in SAD; Mann Whitney test, p = .6216) and then adjusted to reach a trough level of at least 500 mg/dl or higher if a significant infectious burden was still present, considering as IgG target the individual serum IgG trough level required to maintain a patient as infection-free as possible [26].

Fifty-three out of 102 (52.0%) PAD patients and 99 out of 131 (75.6%) SAD patients were naïve to Ig treatment whereas all the others initially had received IVIG and were subsequently switched to subcutaneous formulation. Criteria that drove the choice of switching to SCIG formulation were related to specific patient features (poor venous access, systemic AEs to IVIG, difficult hospital access) and preference (busy schedule, importance of being independent). Different SCIG formulations were used (16% Subcuvia ®16.5% Octanorm ®20% Hizentra ® and 10% facilitated HyQvia ®). Mean duration of SCIG treatment was 73.8 ± 42.4 months (range 12–157 months) in PAD patients and 45.8 ± 23.3 months (range 13–110 months) in SAD patients.

Among 102 PAD patients, 45 (44.1%) received 20% SCIG, 43 (42.1%) 16% or 16.5% SCIG whereas 14 (13.7%) were treated with 10% facilitated SCIG. Of the 131 SAD patients, 75 (57.2%) were treated with 20% SCIG, 54 (41.2%) with 16% SCIG and 2 (1.5%) with 10% facilitated SCIG.

**Fig 2:**
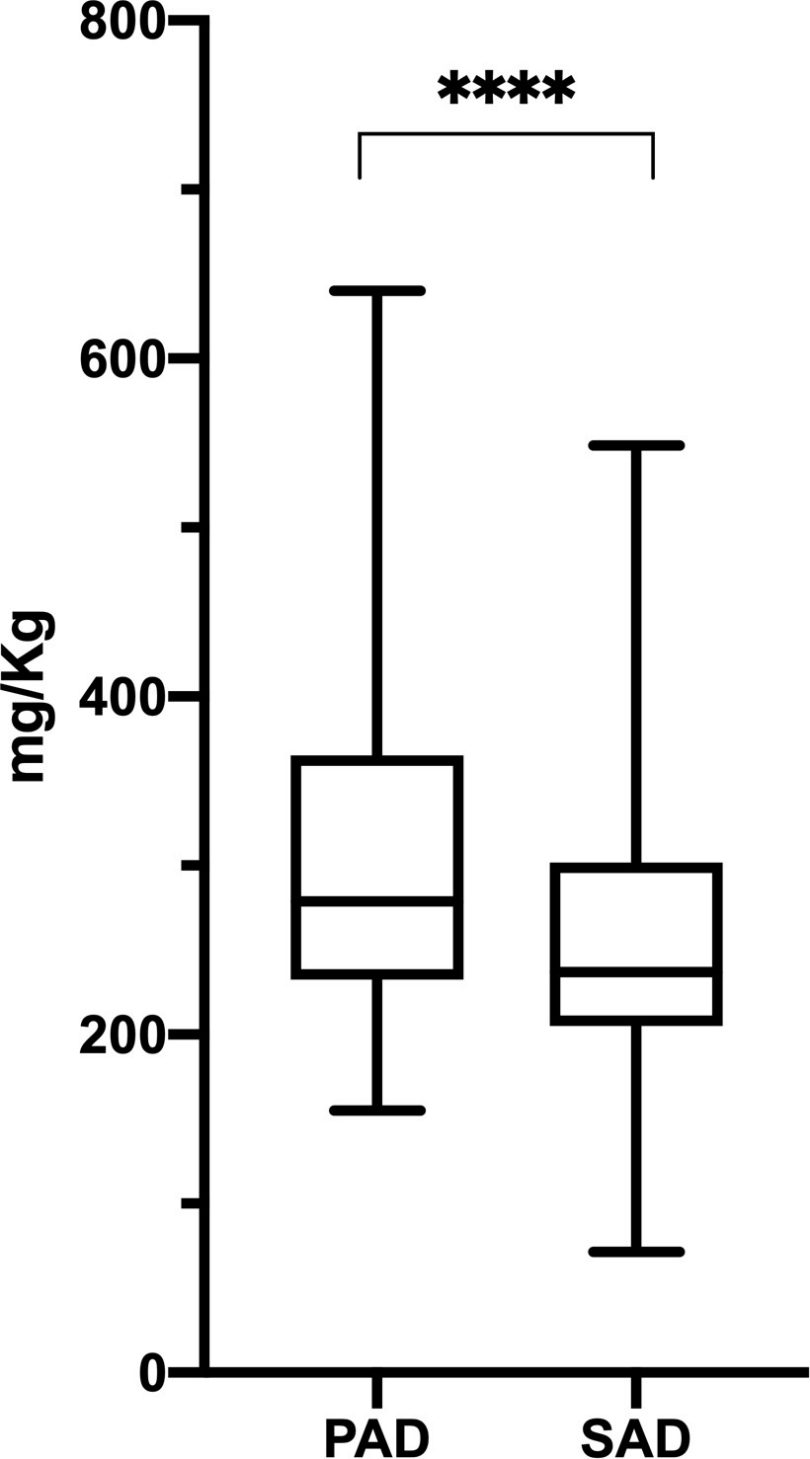
Steady state SCIG dosage in PAD and SAD patients. At the steady state, PAD patients received SCIG at statistic significant higher dose as compared to SAD patients (PAD: 309.31 ± 95.33 mg/kg, range 154.94–640.00 mg/kg; SAD: 251.94 ± 82.20 mg/kg, range 71.42–548.57 mg/kg; p < .0001). Levels of significance for comparison by Mann Whitney test: ns, not significant, *p ≤ .05, **p ≤ .01, ***p ≤ .001, ****p ≤ .0001.

Mean SCIG monthly dosage, at the steady state, was significantly higher in PAD (309.31 ± 95.33 mg/kg, range 154.94–640.00 mg/kg) if compared to SAD (251.94 ± 82.20 mg/kg, range 71.42–548.57 mg/kg) (Mann Whitney test, p < .0001; Fig 2).

When comparing mean initial SCIG dosage against the mean steady state SCIG dosage, we found significant differences both in PAD (Wilcoxon matched-pairs signed rank test, p < .0001) and SAD (Wilcoxon matched-pairs signed rank test, p = .0051) patients. Focusing on the subgroup of PAD patients with CVID and agammaglobulinemia we found a mean steady state SCIG dosage of 323.87 ± 95.99 mg/kg/month (range 213.00–640.00 mg/kg). Between main SAD subgroups (CLL, MM and NHL) there were no statistic significant difference in terms of mean initial [one way ANOVA test, F(2, 108) = 0.7698, p = .4656)] and the steady state [one way ANOVA test, F(2, 108) = 1.016, p = .3654)] SCIG dosage (S1 Fig).

Data analysis on infusion interval was performed by excluding patients treated with facilitated SCIG because of the different pharmacokinetics of this formulation, that allows prolonged interval between-infusions. Mean between-infusions interval in PAD patients was 7.48 ± 1.74 days (range 3–15 days), in SAD patients was 7.73 ± 1.75 days (range 5–15 days). All facilitated SCIG patients received Ig administration every 21 days.

### IgG levels

To address this aim, we excluded PAD patients with isolated IgG subclass deficiency, IgA with IgG subclass deficiency and SPAD (8 patients) and SAD patients with IgG multiple myeloma (13 patients). We also considered only IgRT naïve patients (not receiving IVIG prior SCIG) when calculating IgG level before SCIG.

**Fig 3:**
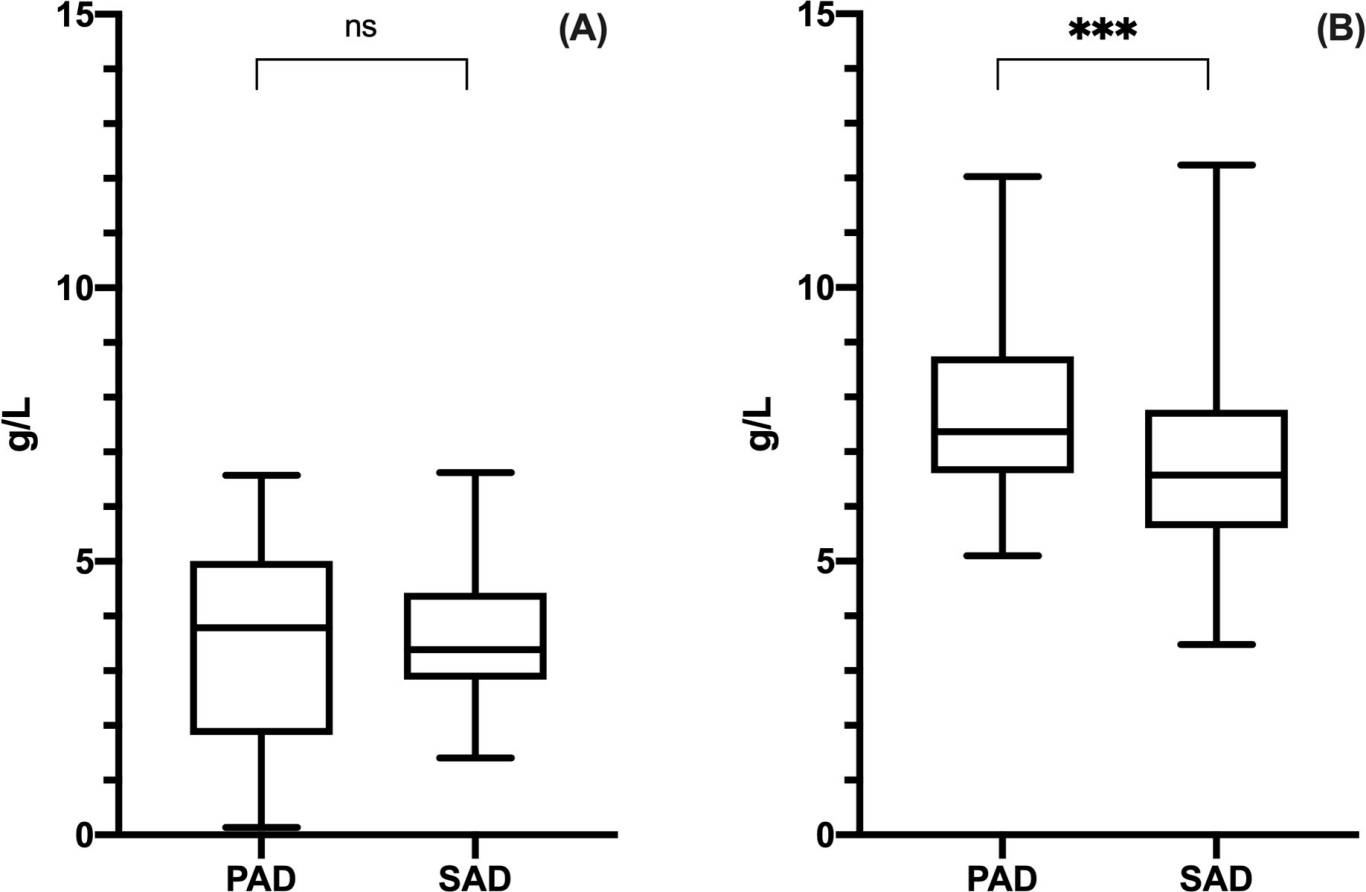
IgG at baseline and trough levels in PAD and SAD patients. Only patients who were naïve to Ig therapy were included in the analysis. There was no statistic significant difference in IgG levels before SCIG (panel A) between PAD and SAD patients, whereas, at the steady state (panel B), PAD patients achieved statistic significant higher trough levels as compared to SAD patients. Levels of significance for comparison by Mann Whitney test (A) and unpaired t test (B): ns, not significant, *p ≤ .05, **p ≤ .01, ***p ≤ .001, ****p ≤ .0001.

Before SCIG, there were no significant differences between PAD and SAD patients in peripheral IgG levels: mean serum IgG were 3.46 ± 1.90 g/L in PAD and 3.68 ± 1.16 g/L in SAD (Mann Whitney test, p = .8767; Fig 3A).

Conversely, we found a statistic significant difference between SAD subgroups (CLL, MM and NHL) in terms of baseline IgG before SCIG RT [Welch’s ANOVA test, F(2.000, 34.83) = 22.42, p < .0001]. Post hoc analysis with Dunnett’s T3 multiple comparisons test indicated that patients with MM had lower initial IgG values (2.75 ± 0.26 g/L), as compared to both NHL [3.46 ± 1.07 g/L, t(33.11) = 3.319, p = .0066] and B-CLL [3.92 ± 1.04 g/L, t(28.61) = 6.622, p < .0001] patients (S2 Fig).

During SCIG therapy, at the steady state, mean IgG trough level was significantly higher in PAD than in SAD patients (7.67 ± 1.45 g/L in PAD, 6.80 ± 1.71 g/L in SAD; unpaired t test, t(199) = 3.808, p = .0002; Fig 3B). Furthermore, there were statistically significant difference in terms of IgG trough levels between SAD subgroups (CLL, MM and NHL) [one way ANOVA test, F(2, 91) = 6.961, p = .0015]. NHL patients achieved the highest values (7.49 ± 1.55 g/L) as compared to CLL (6.26 ± 1.47 g/L) and MM (6.46 ± 1.59 g/L) patients [post hoc analysis with Tukey’s multiple comparisons test indicated q(91) = 5.245, p = .0010 and q(91) = 2.172, p = .2790 vs CLL and vs MM respectively] (S2 Fig).

In PAD cohort there was no statistical correlation between SCIG dosage and IgG trough levels achieved at steady state (r = 0.1234; p = .2414), whereas in SAD patients we observed a significant positive correlation between the aforementioned parameters (r = 0.2365; p = .0099) (S3 Fig).

As expected, SCIG RT had no effects on circulating IgA and IgM (for both PAD and SAD, Ig values before and during SCIG RT are reported in S2 Table). Of note, as compared to IVIG treatment, patients who had been switched to SCIG achieved higher IgG trough levels, in both PAD (4.93 ± 1.53 g/L with IVIG vs 7.52 ± 1.41 g/L with SCIG, Wilcoxon matched-pairs signed rank test, p < .0001; S4A Fig) and SAD cohorts (4.34 ± 1.11 g/L with IVIG vs 6.51 ± 1.73 g/L with SCIG, Wilcoxon matched-pairs signed rank test, p < .0001; S4B Fig).

### Efficacy of SCIG

**Fig 4:**
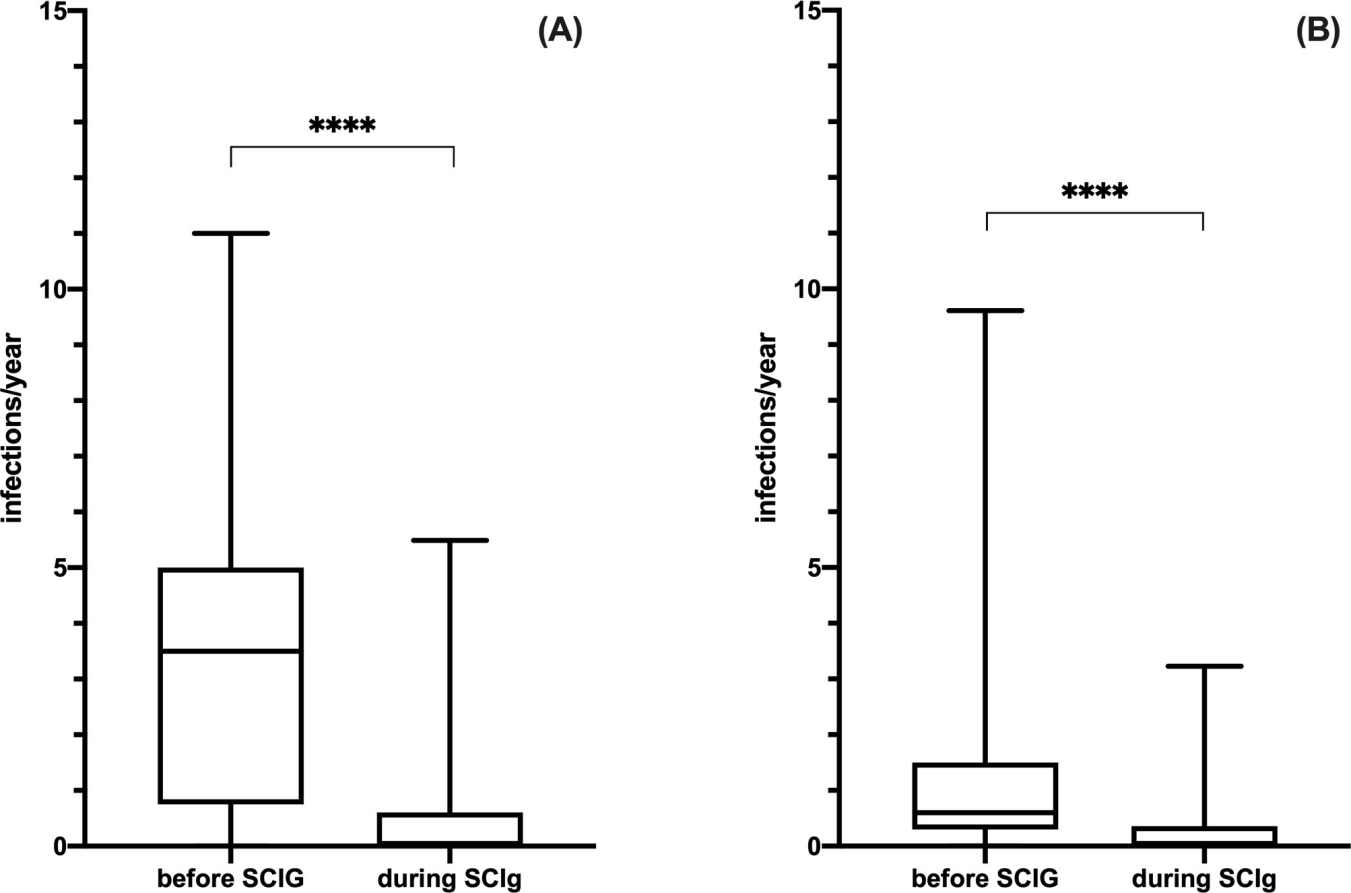
Efficacy of SCIG in PAD and SAD patients. Following SCIG initiation there were a statistic significant decrease in the annual infectious rate in both patients with PAD (panel A) and SAD (panel B). Levels of significance for comparison by Wilcoxon matched-pairs signed rank test: ns, not significant, *p ≤ .05, **p ≤ .01, ***p ≤ .001, ****p ≤ .0001.

We then evaluated the annual infection rate in both cohorts, considering only patients naïve to IgRT (we excluded patients previously treated with IVIG). Forty-four PAD and 85 SAD patients were then available for this analysis. The infection rate in PAD patients before SCIG treatment was 3.69 ± 2.81/year per patient (total of 487 infections, including 40 SBI) and it significantly decreased at 0.52 ± 1.18/year per patient (total of 138 infections, including 1 SBI) during SCIG at the steady state (Wilcoxon matched-pairs signed rank test, p < .0001; Fig 4A). Similarly, in SAD patients 375 infections were recorded before SCIG treatment, including 72 SBI (infection rate of 1.01± 1.38/year per patient), with a drop in infection rate to 0.24 ± 0.44/year per patient during SCIG, with a total of 109 infections including 38 SBI observed (Wilcoxon matched-pairs signed rank test, p < .0001; Fig 4B). There was no statistical difference in terms of infection rate between PAD and SAD during SCIG treatment (Mann Whitney test, p = .9012). By analysing SAD subgroups (CLL, MM and NHL) we did not found any statistic significant difference in terms of infectious rate before [Kruskal-Wallis ANOVA test, H(2) = 0.2529, p = .8812] and during [Kruskal-Wallis ANOVA test, H(2) = 1.329, p = .5146] SCIG RT. Prevalence and types of infections in PAD and SAD patients before SCIG are recapitulated in S3 Table.

### Adverse events and local reactions

A total of 14 PAD patients and 6 SAD patients reported mild side effects during SCIG treatment, defined as local cutaneous rash (10 patients) and pain at infusion site (3 patients), headache (3 patients), mild nausea (3 patients) and chills (2 patients), delayed fever (2 patients, improved during subsequent administrations), skin infection at infusion site (1 patients). No severe adverse events were registered. No discontinuation of SCIG treatment was required due to adverse reactions.

### IgG levels and infection rates after discontinuation of Ig replacement therapy in NHL patients

During the observation period 20 patients with NHL had SCIG replacement therapy suspended. In these patients SCIG treatment had been specifically authorized for a limited amount of months/years by the local Regulatory Agency, since in Italy IgRT for SAD is commonly restricted only to MM, CLL, HSCT and HIV patients. In this group, mean baseline serum IgG were 3.39 ± 1.03 and the annual infection rate before SCIG RT was 2.18 ± 2.82/year per patient. SCIG were administered for a mean of 32.11±16.26 months (range 12–60 months). Globally, the effect of SCIG was significant in modifying both IgG [repeated measures ANOVA test, F(1.505, 27.10) = 38.27, p < .0001] and infectious rate [repeated measures ANOVA test, F(1.830, 29.29) = 5.052, p = .0150].

**Fig 5:**
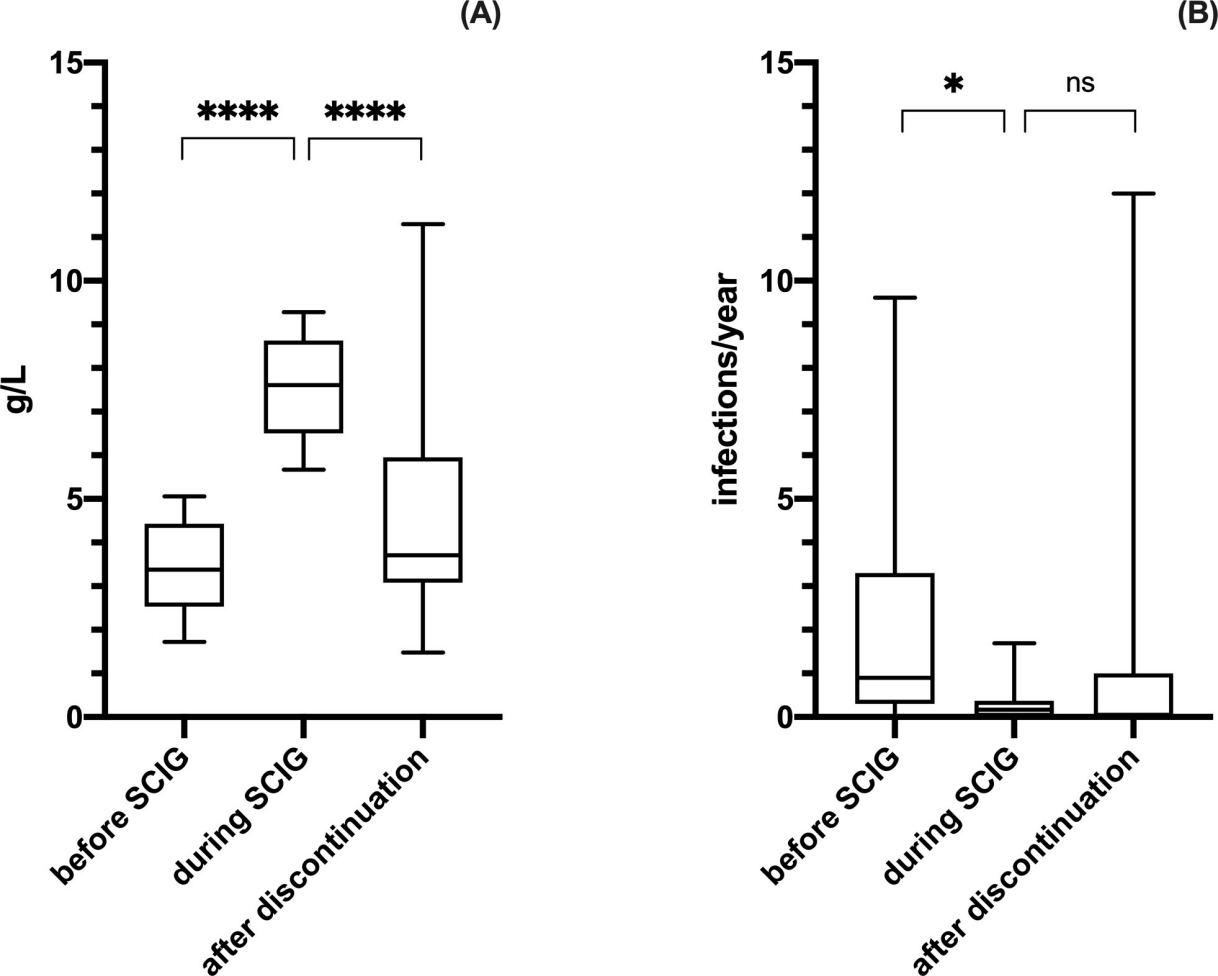
IgG and infectious rate in NHL at baseline, during and after discontinuation of SCIG. There were a statistic significant difference between the three time points both considering IgG levels [repeated measures ANOVA test, F(1.505, 27.10) = 38.27, p < .0001] and infectious rate [repeated measures ANOVA test, F(1.830, 29.29) = 5.052, p = .0150]. Post hoc analysis indicated that SCIG RT significantly increased circulating IgG (panel A) and decreased the annual infectious rate (panel B). Furthermore, following discontinuation of SCIG RT, there was a significant reduction of circulating IgG (panel A) and an increased, although not statistically significant, infection rate (panel B). Levels of significance for comparison by Tukey’s multiple comparisons test: ns, not significant, *p ≤ .05, **p ≤ .01, ***p ≤ .001, ****p ≤ .0001.

IgG trough-levels achieved were 7.53 ± 1.08 g/L and the annual infection rate during SCIG was 0.29 ± 0.46/year per patient (we recorded a total of 24 infections including 2 SBI). After discontinuation of SCIG we found a significant reduction of circulating IgG (mean serum IgG level of 4.58 ± 2.63 g/L; post hoc analysis with Tukey’s multiple comparisons test indicated q(18) = 7.840, p < .0001; Fig 5A) and an increased, although not statistically significant, infection rate (we registered a total of 55 infections including 4 SBI; infection rate 1.59 ± 3.12/year per patient; post hoc analysis with Tukey’s multiple comparisons test indicated q(16) = 2.774, p = .1541; Fig 5B). Mean follow up after discontinuation was 27.62 ± 13.23 months (range 14–62 months). Of note within this group, 3 out of 20 patients showed a progressive spontaneous recovery of IgG production reaching protective levels (mean IgG levels 8.77 ± 2.33 g/L) and registered no infections after discontinuation of SCIG.

## Discussion

Very few studies with small cohorts of patients have addressed the efficacy of SCIG in SAD patients. Hammarström et al first reported, in 1995, a reduction in infections requiring hospital admission and need of antibiotic courses in 17 subjects with chronic lymphoproliferative neoplasms treated with SCIG [27]. Spadaro et al showed that sequential treatment with IVIG (duration of therapy 6 months, mean administered dose of IgG 400 mg/Kg/month) and then with SCIG (mean dose of 100 mg/Kg/week) resulted in comparable protection against infections and higher serum IgG levels with SCIG in a cohort of 14 SAD patients affected by B-CLL and NHL [28]. More recently, a randomized clinical trial demonstrated a significant benefit in terms of annual rate of infections (including severe infections), hospitalization and antibiotic therapy duration, as well as an improvement of HRQoL, in 46 patients affected by MM [29].

Four other studies have clearly shown a significant increase in serum IgG level following SCIG [30–33] (globally 103 patients in 4 SAD cohorts); finally, even if not statistically supported, data from 2 of these 4 reported cohorts suggested in one case equal [30] and in another one better [31] infectious outcome for SAD patients under SCIG treatment as compared to IVIG.

All these previous data, although collected from small cohorts of patients, suggest that SCIG are at least equivalent to IVIG in term of increase of IgG serum levels as well as in reducing infections [27–34]. No conclusive data are available regarding the appropriate SCIG dosage in SAD patients (reported dosages range from 50 to 200 mg/kg/wk) or the optimal IgG trough level [23].

In this study we report the largest SAD cohort treated with SCIG, and we also provide data for a direct comparison with a PAD cohort in terms of effectiveness, dosage and schedule, tolerance, in a single-centre setting. First of all, we confirmed that SCIG therapy in SAD patients is at least as effective as in PAD patients in preventing (non-neutropenic) infections. Even though comparison of IVIG with SCIG was not a study primary endpoint, in the subgroup of patients who had been switched to SCIG we observed higher IgG trough level as compare to the previous IVIG RT. We did not perform any efficacy comparison between IVIG and SCIG in these patients because most of them had regular summer interruptions of therapy during IVIG period (the so called “treatment holidays”) [32].

Secondly, in our cohort of SAD patients, we found that a lower Ig monthly dose was required to achieve similar effectiveness in terms of infection rate reduction, as compared to PAD. The actual dosage in our SAD cohort was close to the lower limit of the previously reported range and significantly lower than what required for PAD patients. This result is particularly valuable when considering that the two cohorts of our study were initially treated with a similar dose of SCIG, that was further adapted to clinical requirements. This is in line with the current concepts of individualization of SCIG treatment [26]. Indeed, our data underscore the importance of targeting a biological effect rather than a specific IgG threshold in SAD patients. We believe that individualizing the dosage based on measured serum IgG levels and the clinical response is preferable to using mean pharmacokinetic parameters [24].

Furthermore, even if PAD patients reached higher IgG trough level as compared to SAD, we found no correlation between SCIG dosage and IgG trough level achieved in PAD, whilst we found a correlation in the SAD cohort. Data from literature report a wide variation in the IgG doses necessary to achieve given IgG levels in different PAD patients [35], but no SCIG dosage-serum IgG correlation data has been previously reported in SAD patients.

A possible explanation for the latter point and for the observed efficacy of SCIG at lower dosage in SAD patients may be found in different underlying disease mechanisms and other patient-dependent factors, like immunity recover after treatment for the underlying disease, shorter history of hypogammaglobulinemia and lower incidence of PAD-related complications as enteropathy and/or bronchiectasis. On the contrary, age does not seem to explain this data, as we found the same difference in SCIG dosage between SAD and PAD patients even when the analysis was performed in subjects younger than 65 yrs old (PAD: 321.2 ± 100.8 mg/kg; SAD: 238.0 ± 71.01 mg/kg, Mann Whitney test, p < .0001).

In terms of tolerance, in agreement with literature data, we observed only mild post-infusion reaction and local site pain as adverse effects in our cohort of SAD patients, making SCIg an attractive option for IgRT in patients with SAD.

We did not perform cost-saving evaluation of SCIG. Nevertheless SCIG therapy has been constantly reported to lower health care costs in PAD patients [20]. A recent cost-utility analysis performed in Australia demonstrated a better health outcomes and cost savings of the home-based SCIG treatment as compared to hospital-based IVIG option in patients with SAD [36].

To date in literature only 43 patients with NHL treated with SCIG have been reported in different cohorts [27, 28, 31, 32, 34, 37]. In Italy as in other countries, IgRT for hematologic malignancies is commonly restricted only to MM, B-CLL or post-HSCT. In our SAD cohort NHL are well represented (34 patients) and allow statistical comparison with other subgroups of hematologic malignancies. As compared to MM and B-CLL we observed a similar infectious rate before and after SCIG treatment in patients affected by NHL. Also, the fact that most of our NHL patients who suspended SCIG RT experienced infectious exacerbations suggests not to restrain indication to SCIG in hematologic neoplasms only to the aforementioned groups, in agreement with the latest EMA guidelines about IVIG use [12].

In conclusion, over the last 20 years, the life expectancy of patients with hematologic malignancies has significantly increased as the result of the availability of novel treatment options including more efficient chemotherapy regimens, monoclonal antibodies, small molecule inhibitors, the increasing use of maintenance or chronic therapy and the better outcome of stem-cell transplantation [38, 39]. In this scenario, infection prevention strategies should also evolve from hospital-based treatments towards individualized home-based therapies that can be adapted to each patient’s lifestyle without requiring a venous access. Thus, SCIG administration in SAD patients seems a logic strategy to pursue, regardless of the underlying hematologic malignancy. Even if not conclusive, given the retrospective- and single-center setting of this study, our data add further strength to the adoption of SCIG in the treatment of SAD. Finally, the current SARS-COV-2 pandemic further highlighted the importance of home-based treatment strategies in immunocompromised patients, especially in countries where IVIG are mandatorily administrated in hospital setting.

## Supporting information

S1 Fig Initial and steady state SCIG dosage in hematologic SAD subgroups. There were no statistic significant difference between initial (panel A) and steady state (panel B) Ig RT dosage in patients with SAD due to hematologic neoplasm (n = 111). (A) One way ANOVA test, F(2, 108) = 0.7698, p = .4656) and (B) One way ANOVA test, F(2, 108) = 1.016, p = .3654). NHL, Non-Hodgkin Lymphoma; MM, Multiple Myeloma; CLL, Chronic Lymphocytic Leukemia. Bars indicated 95%CI of difference.
(TIF)

S2 Fig IgG at baseline and trough levels in hematologic SAD subgroups. There were statistic significant difference between SAD subgroups (CLL, MM and NHL) both in IgG levels at baseline (panel A) [Welch’s ANOVA test, F(2.000, 34.83) = 22.42, p < .0001] and in IgG trough levels (panel B) [one way ANOVA test, F(2, 91) = 6.961, p = .0015]. Post hoc analysis indicated that at baseline (panel A) patients with MM had the lowest initial IgG values, whereas NHL patients achieved the highest IgG trough levels (panel B). NHL, Non-Hodgkin Lymphoma; MM, Multiple Myeloma; CLL, Chronic Lymphocytic Leukemia. Bars indicated 95%CI of difference.
(TIF)

S3 Fig Simple linear regression analysis of SCIG dosage and IgG trough levels in PAD and SAD patients. At the steady state, the covariance between SCIG dosage and IgG trough level was not statistically significant in PAD (panel A) patients (r = 0.1234; p = .2414). On the contrary, in SAD patients (panel B) there were a significant positive correlation between these parameters (r = 0.2365; p = .0099). Dotted lines indicated 95%CI of the best-fit line.
(TIF)

S4 Fig IgG trough levels comparison between IVIG and SCIG in PAD and SAD patients. Patients who had been switched to SCIG achieved higher IgG trough levels as compared to previous treatment with IVIG, in both PAD (panel A) and SAD (panel B) cohorts. Levels of significance for comparison by Wilcoxon matched-pairs signed rank test: ns, not significant, *p ≤ .05, **p ≤ .01, ***p ≤ .001, ****p ≤ .0001.
(TIF)

S1 Table Ig before SCIG and comorbidities in the SAD cohort. (DOCX)

S2 Table Ig before and during SCIG in PAD and SAD. (DOCX)

S3 Table Prevalence and types of infections among PAD and SAD cohorts before SCIG. (DOCX)
