# Supplementary figures and images for: Subcutaneous immunoglobulins replacement therapy in secondary antibody deficiencies: Real life evidence as compared to primary antibody deficiencies
Source: PLoS One. 2021 Mar 4;16(3):e0247717. doi: 10.1371/journal.pone.0247717 (PMC7932095; doi:10.1371/journal.pone.0247717)

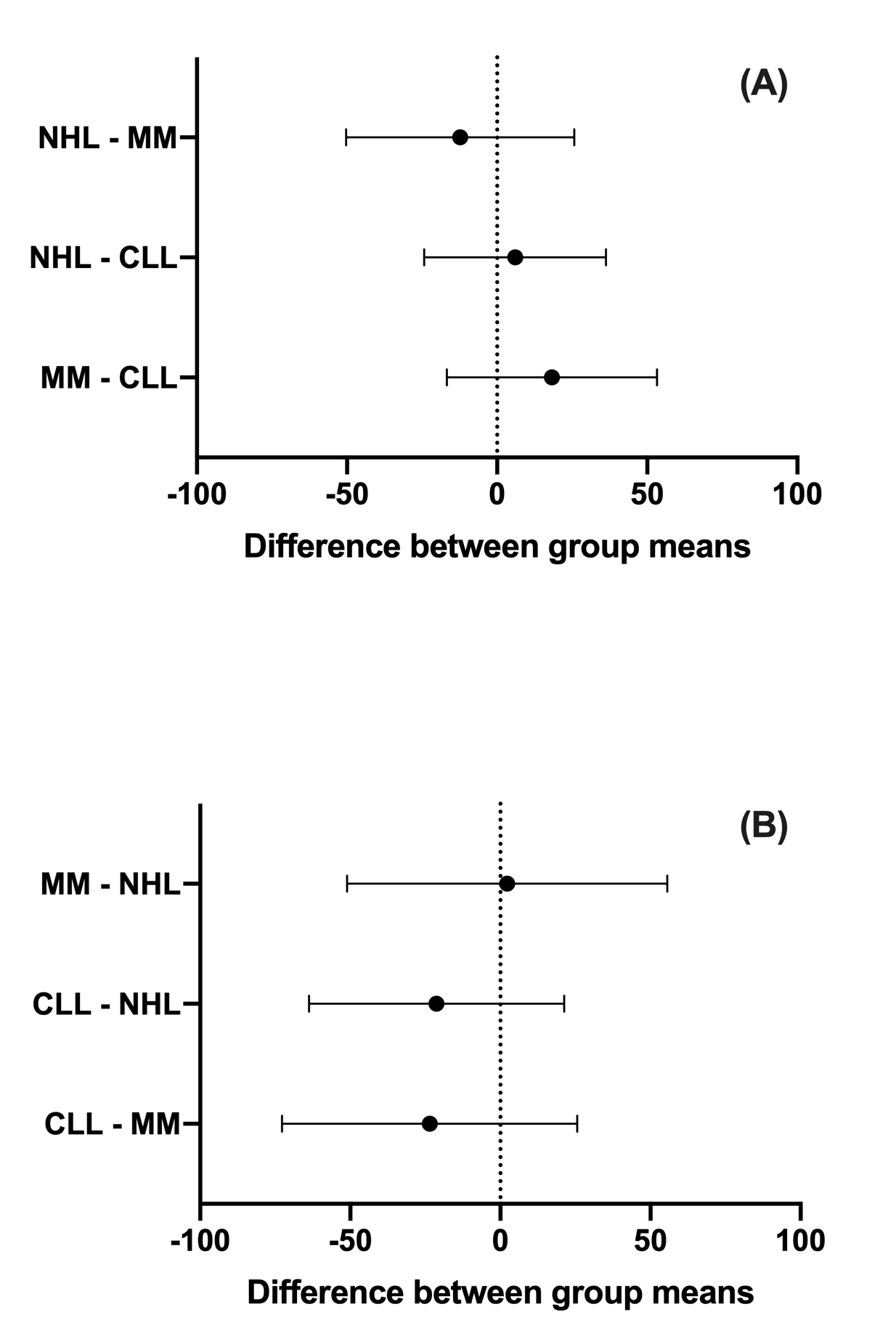

Supplement: S1 Fig — There were no statistic significant difference between initial (panel A) and steady state (panel B) Ig RT dosage in patients with SAD due to hematologic neoplasm (n = 111). (A) One way ANOVA test, F(2, 108) = 0.7698, p = .4656) and (B) One way ANOVA test, F(2, 108) = 1.016, p = .3654). NHL, Non-Hodgkin Lymphoma; MM, Multiple Myeloma; CLL, Chronic Lymphocytic Leukemia. Bars indicated 95%CI of difference. (TIF) [file pone.0247717.s001.tif]

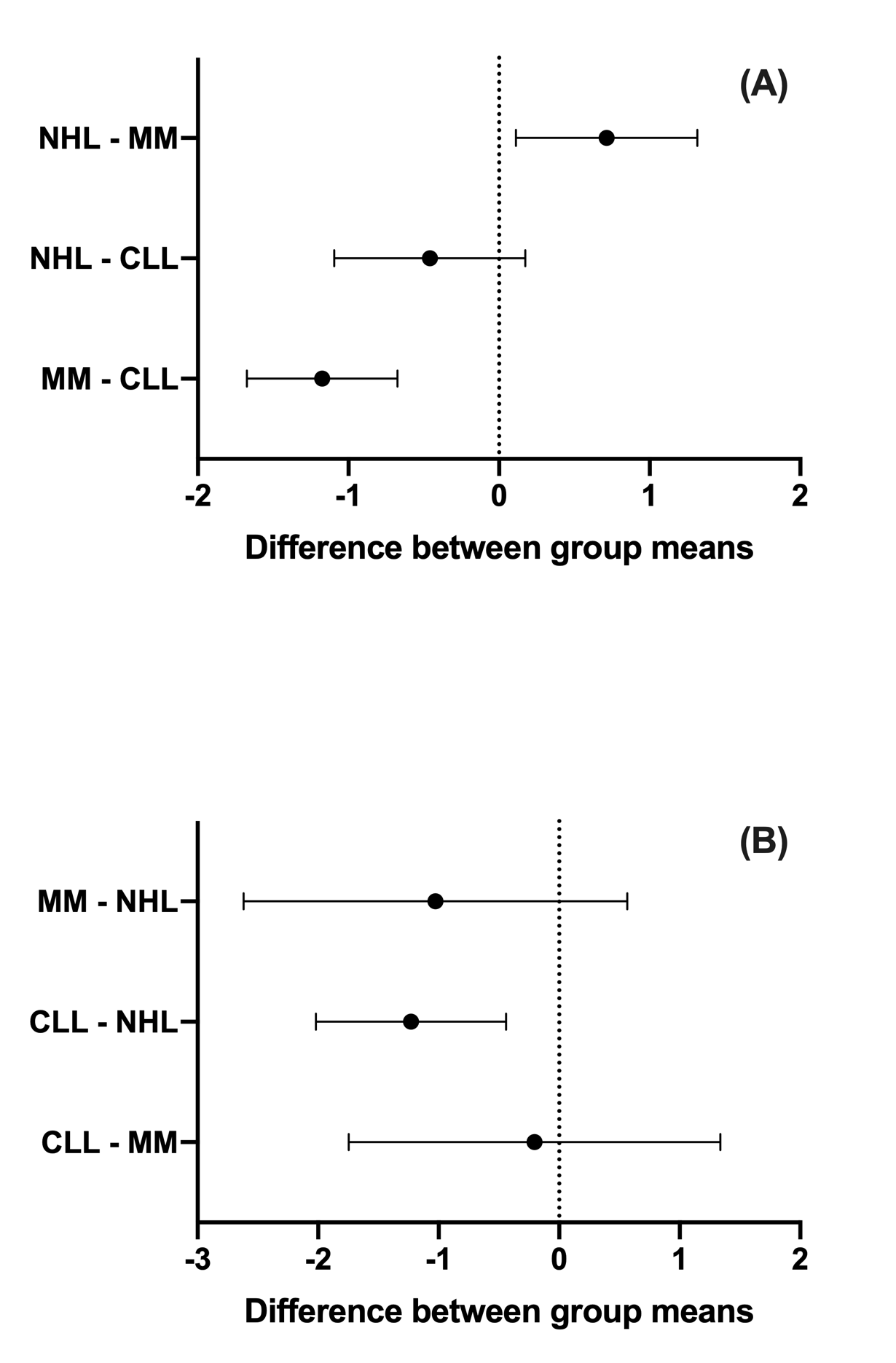

Supplement: S2 Fig — There were statistic significant difference between SAD subgroups (CLL, MM and NHL) both in IgG levels at baseline (panel A) [Welch’s ANOVA test, F(2.000, 34.83) = 22.42, p < .0001] and in IgG trough levels (panel B) [one way ANOVA test, F(2, 91) = 6.961, p = .0015]. Post hoc analysis indicated that at baseline (panel A) patients with MM had the lowest initial IgG values, whereas NHL patients achieved the highest IgG trough levels (panel B). NHL, Non-Hodgkin Lymphoma; MM, Multiple Myeloma; CLL, Chronic Lymphocytic Leukemia. Bars indicated 95%CI of difference. (TIF) [file pone.0247717.s002.tif]

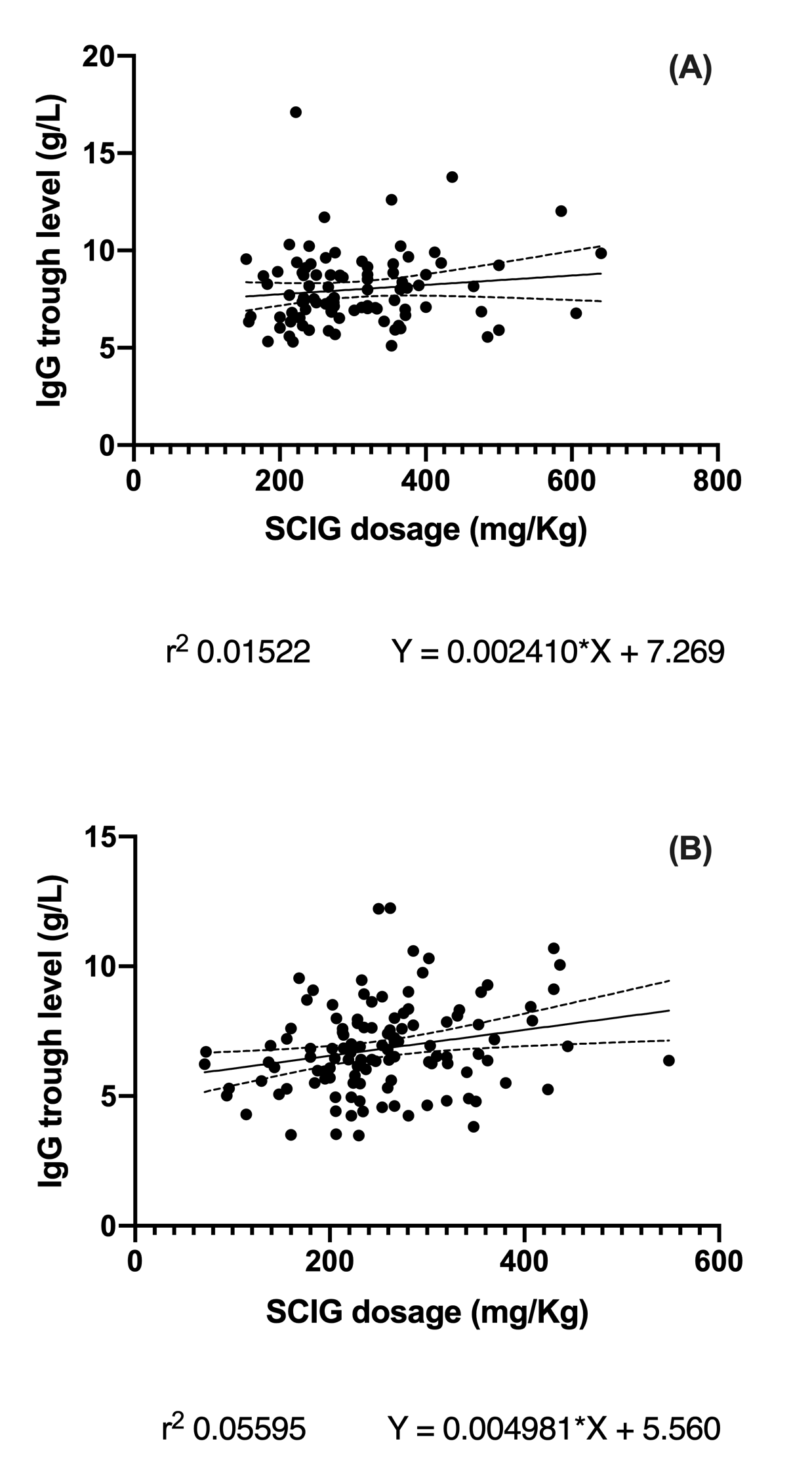

Supplement: S3 Fig — At the steady state, the covariance between SCIG dosage and IgG trough level was not statistically significant in PAD (panel A) patients (r = 0.1234; p = .2414). On the contrary, in SAD patients (panel B) there were a significant positive correlation between these parameters (r = 0.2365; p = .0099). Dotted lines indicated 95%CI of the best-fit line. (TIF) [file pone.0247717.s003.tif]

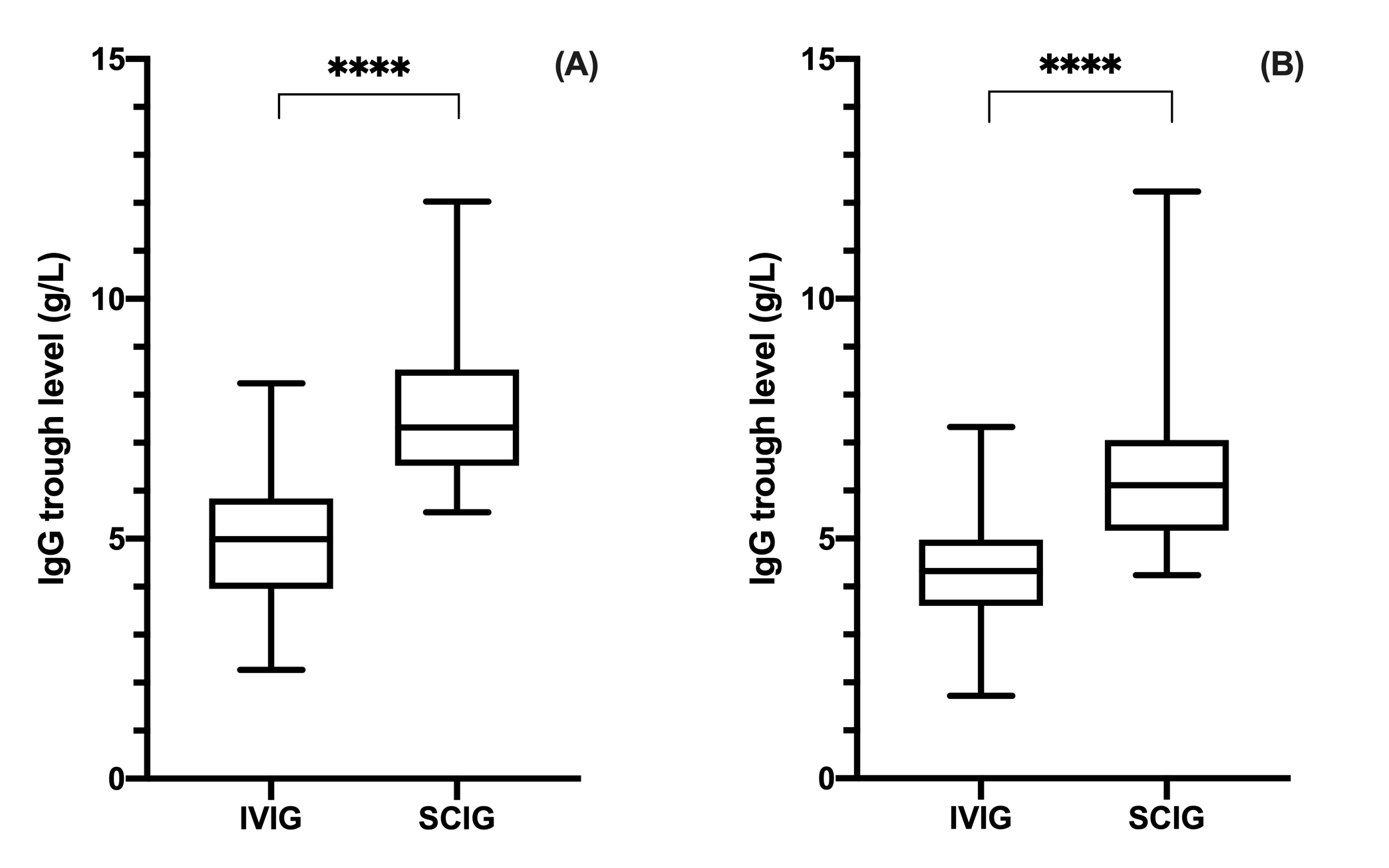

Supplement: S4 Fig — Patients who had been switched to SCIG achieved higher IgG trough levels as compared to previous treatment with IVIG, in both PAD (panel A) and SAD (panel B) cohorts. Levels of significance for comparison by Wilcoxon matched-pairs signed rank test: ns, not significant, *p ≤ .05, **p ≤ .01, ***p ≤ .001, ****p ≤ .0001. (TIF) [file pone.0247717.s004.tif]
