# Supplementary material for: Subcutaneous immunoglobulins replacement therapy in secondary antibody deficiencies: Real life evidence as compared to primary antibody deficiencies
Source: PLoS One. 2021 Mar 4;16(3):e0247717. doi: 10.1371/journal.pone.0247717 (PMC7932095; doi:10.1371/journal.pone.0247717)
Supplement: S1 Table — (DOCX) [file pone.0247717.s005.docx]

**S1 Table.** **Ig before SCIG and comorbidities in the SAD cohort.**

| **Baseline immunoglobulins and comorbidities** | **SAD, n = 131** | **MM, n = 22** | **CLL, n = 55** | **NHL, n = 34** | **Others, n = 20** |
| --- | --- | --- | --- | --- | --- |
| **Immunoglobulins** |  |  |  |  |  |
| IgG (g/L), Mean ± SD | 3,68 ± 1,16 | 2,75 ± 0,26 | 3,92 ± 1,04 | 3,46 ± 1,07 | 4,20 ± 1,51 |
| IgA (g/L), Mean ± SD | 0,50 ± 0,75 | 0,50 ± 0,50 | 0,52 ± 0,95 | 0,42 ± 0,29 | 0,50 ± 0,23 |
| IgM (g/L), Mean ± SD | 0,31 ± 0,42 | 0,15 ± 0,08 | 0,31 ± 0,40 | 0,26 ± 0,21 | 0,92 ± 1,02 |
| **Comorbidities** |  |  |  |  |  |
| ^a^ Chronic Lung Disease, n (%) | 37 (28,3) | 5 (22,7) | 22 (40) | 5 (14,7) | 5 (25) |
| CKD, n (%) | 22 (16,8) | 6 (27,3) | 7 (12,7) | 5 (14,7) | 4 (20) |
| Malabsorption, n (%) | 0 (0) | 0 (0) | 0 (0) | 0 (0) | 0 (0) |
| Splenectomy, n (%) | 4 (3) | 0 (0) | 3 (5,4) | 0 (0) | 1 (5) |
| Diabetes, n (%) | 13 (9,9) | 5 (22,7) | 6 (11) | 1 (3) | 1 (5) |
| Cytopenia, n (%) | 20 (15,3) | 11 (50) | 1 (1,8) | 4 (11,8) | 4 (20) |

CKD = Chronic Kidney Disease

^a^ Chronic Lung Disease was defined as the presence of asthma, chronic obstructive pulmonary disease, interstitial lung disease, bronchiectasis or alpha1-antitrypsin deficiency.
