# Supplementary material for: Subcutaneous immunoglobulins replacement therapy in secondary antibody deficiencies: Real life evidence as compared to primary antibody deficiencies
Source: PLoS One. 2021 Mar 4;16(3):e0247717. doi: 10.1371/journal.pone.0247717 (PMC7932095; doi:10.1371/journal.pone.0247717)
Supplement: S2 Table — (DOCX) [file pone.0247717.s006.docx]

**S2 Table. Ig before and during SCIG in PAD and SAD**

|  | **IgG (g/L), Mean ± SD** | **IgA (g/L), Mean ± SD** | **IgM (g/L), Mean ± SD** |
| --- | --- | --- | --- |
| **PAD cohort** |  |  |  |
| before SCIG | 3,46 ± 1,90 | 0,45 ± 0,62 | 0,42 ± 0,45 |
| during SCIG | 7,67 ± 1,45 | 0,46 ± 0,63 | 0,47 ± 0,46 |
| **SAD cohort** |  |  |  |
| before SCIG | 3,68 ± 1,16 | 0,50 ± 0,75 | 0,31 ± 0,42 |
| during SCIG | 6,80 ± 1,71 | 0,54 ± 0,80 | 0,52 ± 1,15 |
