# Supplementary material for: Subcutaneous immunoglobulins replacement therapy in secondary antibody deficiencies: Real life evidence as compared to primary antibody deficiencies
Source: PLoS One. 2021 Mar 4;16(3):e0247717. doi: 10.1371/journal.pone.0247717 (PMC7932095; doi:10.1371/journal.pone.0247717)
Supplement: S3 Table — (DOCX) [file pone.0247717.s007.docx]

**S3 Table.** **Prevalence and types of infections among PAD and SAD cohorts before SCIG.**

|  | **PAD, n (%)** | **SAD, n (%)** |
| --- | --- | --- |
| **Total infections** | 487 (100) | 375 (100) |
| **Type of infections** |  |  |
| Minor infections | 447 (91,8) | 303 (80,8) |
| Upper respiratory tract infection | 238 (53,2) | 195 (64,4) |
| Sinusitis | 110 (24,6) | 39 (12,9) |
| Bacterial skin infection | 13 (2,9) | 17 (5,6) |
| Bacterial stomatitis | 4 (0,9) | 1 (0,3) |
| Lower urinary tract infection | 28 (6,3) | 34 (11,2) |
| Herpes virus infection | 5 (1,1) | 9 (3,0) |
| Gastroenteritis | 42 (9,4) | 5 (1,6) |
| Fever of unknown origin | 7 (1,6) | 3 (1,0) |
| Serious bacterial infections | 40 (8,2) | 72 (19,2) |
| Pneumonia | 27 (67,5) | 52 (72,2) |
| Sepsis | 12 (30) | 11 (15,3) |
| Meningitis | 1 (2,5) | 2 (2,8) |
| Endocarditis | 0 (0) | 2 (2,8) |
| Osteomyelitis | 0 (0) | 1 (1,4) |
| Septic arthritis | 0 (0) | 1 (1,4) |
| Visceral abscess | 0 (0) | 3 (4,2) |
